# (2′-Amino-4,4′-bi-1,3-thia­zol-2-aminium-κ^2^ *N*,*N*′)aqua­[citrato(4−)-κ^3^ *O*,*O*′,*O*′′)chromium(III) dihydrate

**DOI:** 10.1107/S1600536809005868

**Published:** 2009-02-25

**Authors:** Bing-Xin Liu, Mei Du, Guang-Hua Chen, Xiao-Yuan Sun

**Affiliations:** a Department of Chemistry, Shanghai University, Shanghai 200444, People’s Republic of China; b Department of Chemistry, Anshan Teachers College, Anshan 114005, People’s Republic of China

## Abstract

In the title compound, [Cr(C_6_H_7_N_4_S_2_)(C_6_H_4_O_7_)(H_2_O)]·2H_2_O, the Cr^III^ atom is in a distorted octa­hedral environment, coordinated by one water mol­ecule, two N atoms from a protonated diamino­bithia­zole ligand and three O atoms from a citrate(4−) anion. The complex is zwitterionic, with the H atom from the uncoordinated carboxyl­ate group of the citrate anion transferred to one amino group of the diamino­bithia­zole ligand. O—H⋯O and N—H⋯O hydrogen bonds link the complexes into layers including the two uncoordinated water mol­ecules.

## Related literature

For general background concerning transition-metal complexes of diamino­bithia­zole, see: Waring (1981 ▶); Fisher *et al.* (1985 ▶). For related structures, see: Liu & Xu (2004 ▶); Luo *et al.* (2004 ▶); Liu *et al.* (2004 ▶, 2006 ▶).
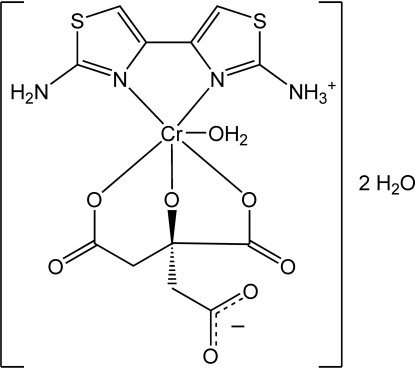


## Experimental

### 

#### Crystal data

[Cr(C_6_H_7_N_4_S_2_)(C_6_H_4_O_7_)(H_2_O)]·2H_2_O
*M* *_r_* = 493.42
Triclinic, 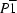

*a* = 7.7438 (15) Å
*b* = 11.193 (2) Å
*c* = 12.057 (2) Å
α = 72.350 (3)°
β = 77.090 (2)°
γ = 82.273 (3)°
*V* = 968.2 (3) Å^3^
*Z* = 2
Mo *K*α radiation
μ = 0.87 mm^−1^
*T* = 295 K
0.25 × 0.20 × 0.15 mm

#### Data collection

Bruker APEXII CCD diffractometer
Absorption correction: multi-scan (*SADABS*; Sheldrick, 1996 ▶) *T*_min_ = 0.810, *T*_max_ = 0.870
5085 measured reflections
3373 independent reflections
2273 reflections with *I* > 2σ(*I*)
*R*_int_ = 0.034

#### Refinement

*R*[*F* ^2^ > 2σ(*F* ^2^)] = 0.060
*wR*(*F* ^2^) = 0.169
*S* = 1.06
3373 reflections
263 parameters
H-atom parameters constrained
Δρ_max_ = 0.89 e Å^−3^
Δρ_min_ = −0.64 e Å^−3^

### 

Data collection: *APEX2* (Bruker, 2004 ▶); cell refinement: *SAINT* (Bruker, 2004 ▶); data reduction: *SAINT*; program(s) used to solve structure: *SIR92* (Altomare *et al.*, 1993 ▶); program(s) used to refine structure: *SHELXL97* (Sheldrick, 2008 ▶); molecular graphics: *ORTEP-3 for Windows* (Farrugia, 1997 ▶); software used to prepare material for publication: *WinGX* (Farrugia, 1999 ▶).

## Supplementary Material

Crystal structure: contains datablocks I, global. DOI: 10.1107/S1600536809005868/bi2321sup1.cif

Structure factors: contains datablocks I. DOI: 10.1107/S1600536809005868/bi2321Isup2.hkl

Additional supplementary materials:  crystallographic information; 3D view; checkCIF report

## Figures and Tables

**Table 1:** Hydrogen-bond geometry (Å, °)

*D*—H⋯*A*	*D*—H	H⋯*A*	*D*⋯*A*	*D*—H⋯*A*
O1—H1*A*⋯O2*W*	0.85	1.85	2.689 (7)	171
O1—H1*B*⋯O12^i^	0.86	1.76	2.597 (5)	164
O1*W*—H1*WB*⋯O15^ii^	0.84	1.92	2.718 (7)	159
O1*W*—H1*WA*⋯O14	0.97	1.83	2.782 (7)	168
O2*W*—H2*WA*⋯O13^iii^	0.97	1.85	2.781 (6)	160
O2*W*—H2*WB*⋯O16^iv^	0.87	1.89	2.690 (8)	152
N22—H22*A*⋯O15	0.85	2.12	2.942 (6)	162
N22—H22*B*⋯O1*W*^v^	0.84	2.15	2.867 (7)	144
N24—H24*A*⋯O11	0.89	2.05	2.864 (6)	152
N24—H24*B*⋯O14^vi^	0.89	2.11	2.901 (6)	148

Symmetry codes: (i) 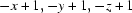; (ii) 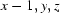; (iii) 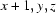; (iv) 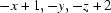; (v) 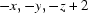; (vi) 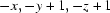.

**Fig. 1.**
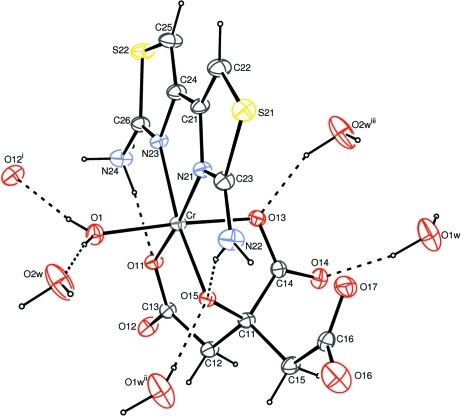
The molecular structure with 30% probability displacement ellipsoids for non-H atoms. Dashed lines indicate hydrogen bonds. [Symmetry codes: (i) -x + 1, -y + 1, -z + 1; (ii) x + 1, y, z; (iii) x - 1, y, z].

**Fig. 2.**
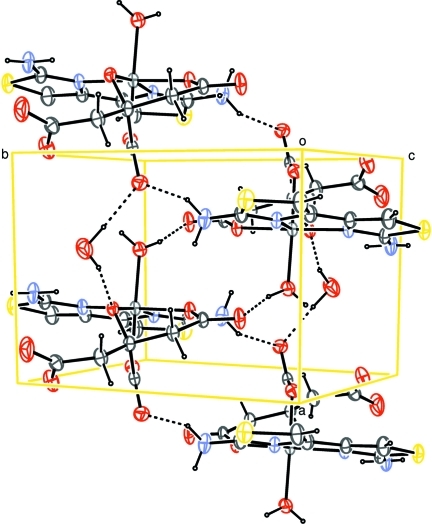
Packing diagram showing hydrogen bonding between CrIII complex molecules.

## supplementary crystallographic information

### Comment

Transition-metal complexes of 2,2'-diamino-4,4'-bi-1,3-thiazole (DABT) have shown potential application in some fields: for example, a Co^II^ complex and a Ni^II^ complex with DABT have been found to be effective inhibitors of DNA synthesis of tumor cell (Waring, 1981; Fisher *et al.*, 1985). The title Cr^III^ complex forms part of a series of structural investigations of metal complexes with DABT.

The complex (Figure 1) displays a distorted octahedral coordination geometry formed by a protonated DABT molecule, one citrate anion and one coordinated water molecule. The citrate anion coordinates to Cr^III^ in a tridentate manner through O atoms of two carboxyl groups and one hydroxyl group. The complex is zwitterionic, with the H atom from the non-coordinated carboxyl group of the citrate anion transferred to one of the amino groups of the DABT ligand.

The thiazole rings of DABT are approximately coplanar (dihedal angle 3.3 (3)°). The C—N(thiazole ring) [N23—C26 = 1.321 (6), N21—C23 = 1.328 (6) Å] and C—N(amino) bonds [N24—C26 = 1.328 (7), N22—C23 1.314 (7) Å] are approximately equal, suggesting the existence of electron delocalization between amino group and thiazole rings. The central C—C bond distance of 1.456 (7) Å in the DABT ligand suggests that a C—C single bond is formed between the *sp*^2^ hybridised C atoms of the thiazole rings.

Extensive hydrogen bonding occurs in the crystal. All lattice water molecules are involved in hydrogen bonding as shown in Fig. 1 and Fig. 2. The amino groups of DABT are hydrogen bonded to adjacent coordinated oxygen or lattice water (Fig. 1) *via* N—H···O hydrogen bonds to form a layered structure parallel to the (011) planes.

### Experimental

An ethanol solution (20 ml) containing DABT (0.20 g 1 mmol) and CrCl_3_^.^6H_2_O (0.27 g 1 mmol) was mixed with an aqueous solution (10 ml) of citric acid (0.19 g 1 mmol) and NaOH (0.12 g 3 mmol). The mixture was refluxed for 6 h. After cooling to room temperature the solution was filtered. Single crystals were obtained from the filtrate after 2 d.

### Refinement

H atoms on C atoms were placed in calculated positions, with C—H = 0.93 Å (aromatic) or C—H = 0.97 (methylene), and were included in the final cycles of refinement in riding mode with *U*_iso_(H) = 1.2*U*_eq_(C). H atoms of the amino group of DABT, coordinated water and lattice water were located in difference Fourier maps and included in the final cycles of refinement in riding mode with *U*_iso_(H) = 1.2*U*_eq_(N) and 1.5*U*_eq_(O). H atoms of the ammonium group were visible in a difference Fourier map, but placed geometrically and allowed to rotate about the C—N bond during the final cycles of refinement.

### Crystal data

**Table:**

[Cr(C_6_H_7_N_4_S_2_)(C_6_H_4_O_7_)(H_2_O)]·2H_2_O	*Z* = 2
*M**_r_* = 493.42	*F*(000) = 506
Triclinic, *P*1	*D*_x_ = 1.693 Mg m^−^^3^
Hall symbol: -P 1	Mo *K*α radiation, λ = 0.71073 Å
*a* = 7.7438 (15) Å	Cell parameters from 3270 reflections
*b* = 11.193 (2) Å	θ = 2.0–25.0°
*c* = 12.057 (2) Å	µ = 0.87 mm^−^^1^
α = 72.350 (3)°	*T* = 295 K
β = 77.090 (2)°	Prism, red
γ = 82.273 (3)°	0.25 × 0.20 × 0.15 mm
*V* = 968.2 (3) Å^3^	

### Data collection

**Table:**

Bruker APEXII CCD diffractometer	3373 independent reflections
Radiation source: fine-focus sealed tube	2273 reflections with *I* > 2σ(*I*)
graphite	*R*_int_ = 0.034
ω scans	θ_max_ = 25.0°, θ_min_ = 1.9°
Absorption correction: multi-scan (*SADABS*; Sheldrick, 1996)	*h* = −9→9
*T*_min_ = 0.810, *T*_max_ = 0.870	*k* = −10→13
5085 measured reflections	*l* = −10→14

### Refinement

**Table:**

Refinement on *F*^2^	Primary atom site location: structure-invariant direct methods
Least-squares matrix: full	Secondary atom site location: difference Fourier map
*R*[*F*^2^ > 2σ(*F*^2^)] = 0.060	Hydrogen site location: inferred from neighbouring sites
*wR*(*F*^2^) = 0.169	H-atom parameters constrained
*S* = 1.06	*w* = 1/[σ^2^(*F*_o_^2^) + (0.076*P*)^2^ + 0.5014*P*] where *P* = (*F*_o_^2^ + 2*F*_c_^2^)/3
3373 reflections	(Δ/σ)_max_ < 0.001
263 parameters	Δρ_max_ = 0.89 e Å^−^^3^
0 restraints	Δρ_min_ = −0.64 e Å^−^^3^

### Special details

**Table:**

Geometry. All e.s.d.'s (except the e.s.d. in the dihedral angle between two l.s. planes) are estimated using the full covariance matrix. The cell e.s.d.'s are taken into account individually in the estimation of e.s.d.'s in distances, angles and torsion angles; correlations between e.s.d.'s in cell parameters are only used when they are defined by crystal symmetry. An approximate (isotropic) treatment of cell e.s.d.'s is used for estimating e.s.d.'s involving l.s. planes.
Refinement. Refinement of *F*^2^ against ALL reflections. The weighted *R*-factor *wR* and goodness of fit *S* are based on *F*^2^, conventional *R*-factors *R* are based on *F*, with *F* set to zero for negative *F*^2^. The threshold expression of *F*^2^ > σ(*F*^2^) is used only for calculating *R*-factors(gt) *etc*. and is not relevant to the choice of reflections for refinement. *R*-factors based on *F*^2^ are statistically about twice as large as those based on *F*, and *R*- factors based on ALL data will be even larger.

### Fractional atomic coordinates and isotropic or equivalent isotropic displacement parameters (Å^2^)

**Table:**

	*x*	*y*	*z*	*U*_iso_*/*U*_eq_	
Cr	0.32133 (11)	0.27404 (7)	0.65363 (7)	0.0290 (3)	
O1	0.5836 (5)	0.2674 (3)	0.5998 (3)	0.0418 (10)	
H1A	0.6573	0.2155	0.6353	0.063*	
H1B	0.6172	0.3088	0.5271	0.063*	
O11	0.3172 (5)	0.4582 (3)	0.6053 (3)	0.0352 (9)	
O12	0.2939 (6)	0.6489 (3)	0.6263 (3)	0.0494 (11)	
O13	0.0634 (5)	0.2891 (3)	0.7116 (3)	0.0360 (9)	
O14	−0.1244 (5)	0.3834 (4)	0.8348 (3)	0.0443 (10)	
O15	0.3302 (4)	0.2688 (3)	0.8123 (3)	0.0306 (8)	
O16	0.1701 (7)	0.1124 (5)	1.1262 (5)	0.0875 (19)	
O17	0.0189 (6)	0.1239 (4)	0.9877 (4)	0.0590 (12)	
N21	0.3201 (6)	0.0846 (4)	0.6822 (4)	0.0326 (10)	
N22	0.3901 (7)	−0.0065 (4)	0.8689 (4)	0.0515 (14)	
H22A	0.3982	0.0724	0.8481	0.062*	
H22B	0.3924	−0.0452	0.9399	0.062*	
N23	0.2795 (6)	0.2683 (4)	0.4916 (4)	0.0327 (10)	
N24	0.2762 (7)	0.4763 (4)	0.3704 (4)	0.0496 (13)	
H24A	0.2657	0.4952	0.4383	0.060*	
H24B	0.1941	0.5221	0.3310	0.060*	
H24C	0.3837	0.4931	0.3267	0.060*	
C11	0.1840 (7)	0.3480 (5)	0.8508 (5)	0.0319 (12)	
C12	0.2369 (8)	0.4857 (5)	0.8038 (5)	0.0369 (13)	
H12A	0.1390	0.5393	0.8324	0.044*	
H12B	0.3377	0.4920	0.8366	0.044*	
C13	0.2847 (7)	0.5345 (5)	0.6699 (5)	0.0334 (13)	
C14	0.0249 (7)	0.3398 (5)	0.7979 (5)	0.0328 (13)	
C15	0.1365 (8)	0.3097 (5)	0.9857 (5)	0.0418 (14)	
H15A	0.2314	0.3291	1.0166	0.050*	
H15B	0.0293	0.3583	1.0106	0.050*	
C16	0.1074 (8)	0.1713 (5)	1.0371 (5)	0.0407 (14)	
C21	0.2760 (7)	0.0473 (5)	0.5913 (5)	0.0342 (13)	
C22	0.2699 (9)	−0.0764 (5)	0.6137 (5)	0.0492 (16)	
H22	0.2430	−0.1147	0.5617	0.059*	
C23	0.3463 (8)	−0.0147 (5)	0.7724 (5)	0.0377 (13)	
C24	0.2508 (8)	0.1495 (5)	0.4864 (5)	0.0382 (14)	
C25	0.2033 (9)	0.1473 (5)	0.3873 (5)	0.0497 (16)	
H25	0.1784	0.0753	0.3725	0.060*	
C26	0.2541 (8)	0.3550 (5)	0.3931 (5)	0.0380 (13)	
S21	0.3177 (2)	−0.15618 (13)	0.75051 (14)	0.0481 (4)	
S22	0.1944 (2)	0.29610 (15)	0.29087 (14)	0.0514 (5)	
O1W	−0.4052 (7)	0.2295 (5)	0.9370 (5)	0.092 (2)	
H1WB	−0.4859	0.2609	0.8983	0.139*	
H1WA	−0.3124	0.2878	0.9110	0.139*	
O2W	0.8198 (6)	0.1215 (4)	0.7251 (6)	0.092 (2)	
H2WA	0.9168	0.1753	0.7039	0.138*	
H2WB	0.8400	0.0585	0.7851	0.138*	

### Atomic displacement parameters (Å^2^)

**Table:**

	*U*^11^	*U*^22^	*U*^33^	*U*^12^	*U*^13^	*U*^23^
Cr	0.0396 (5)	0.0212 (4)	0.0259 (5)	−0.0049 (4)	−0.0082 (4)	−0.0039 (3)
O1	0.041 (2)	0.042 (2)	0.036 (2)	−0.0051 (18)	−0.0061 (18)	−0.0015 (18)
O11	0.054 (2)	0.0220 (18)	0.029 (2)	−0.0064 (16)	−0.0078 (17)	−0.0050 (15)
O12	0.080 (3)	0.021 (2)	0.038 (2)	−0.0078 (19)	0.002 (2)	−0.0038 (17)
O13	0.039 (2)	0.037 (2)	0.034 (2)	−0.0071 (17)	−0.0090 (17)	−0.0099 (17)
O14	0.039 (2)	0.047 (2)	0.042 (2)	−0.0004 (19)	−0.0046 (19)	−0.0078 (19)
O15	0.036 (2)	0.0276 (18)	0.026 (2)	−0.0044 (16)	−0.0078 (16)	−0.0032 (15)
O16	0.103 (4)	0.067 (3)	0.077 (4)	−0.022 (3)	−0.049 (3)	0.031 (3)
O17	0.081 (3)	0.045 (3)	0.052 (3)	−0.027 (2)	−0.011 (2)	−0.006 (2)
N21	0.046 (3)	0.022 (2)	0.029 (3)	−0.0060 (19)	−0.008 (2)	−0.0043 (18)
N22	0.080 (4)	0.034 (3)	0.037 (3)	−0.008 (3)	−0.024 (3)	0.004 (2)
N23	0.047 (3)	0.024 (2)	0.026 (2)	−0.002 (2)	−0.008 (2)	−0.0060 (18)
N24	0.076 (4)	0.033 (3)	0.041 (3)	−0.003 (2)	−0.025 (3)	−0.002 (2)
C11	0.041 (3)	0.025 (3)	0.030 (3)	−0.004 (2)	−0.008 (2)	−0.007 (2)
C12	0.052 (4)	0.027 (3)	0.031 (3)	−0.007 (3)	−0.007 (3)	−0.007 (2)
C13	0.043 (3)	0.021 (3)	0.035 (3)	−0.005 (2)	−0.005 (3)	−0.007 (2)
C14	0.040 (3)	0.024 (3)	0.030 (3)	−0.007 (2)	−0.005 (2)	−0.001 (2)
C15	0.058 (4)	0.036 (3)	0.030 (3)	−0.010 (3)	−0.005 (3)	−0.006 (2)
C16	0.045 (3)	0.043 (3)	0.029 (3)	−0.008 (3)	−0.002 (3)	−0.005 (3)
C21	0.047 (3)	0.024 (3)	0.033 (3)	−0.002 (2)	−0.005 (2)	−0.012 (2)
C22	0.072 (4)	0.033 (3)	0.047 (4)	−0.007 (3)	−0.011 (3)	−0.016 (3)
C23	0.049 (4)	0.022 (3)	0.040 (4)	−0.001 (2)	−0.008 (3)	−0.008 (2)
C24	0.051 (4)	0.030 (3)	0.037 (3)	−0.005 (3)	−0.009 (3)	−0.012 (2)
C25	0.080 (5)	0.034 (3)	0.043 (4)	−0.014 (3)	−0.020 (3)	−0.012 (3)
C26	0.051 (4)	0.034 (3)	0.030 (3)	−0.006 (3)	−0.012 (3)	−0.006 (2)
S21	0.0678 (11)	0.0221 (7)	0.0491 (10)	−0.0039 (7)	−0.0075 (8)	−0.0049 (6)
S22	0.0758 (12)	0.0484 (10)	0.0363 (9)	−0.0050 (8)	−0.0240 (8)	−0.0117 (7)
O1W	0.072 (3)	0.090 (4)	0.092 (4)	−0.034 (3)	−0.045 (3)	0.043 (3)
O2W	0.060 (3)	0.045 (3)	0.164 (6)	−0.012 (2)	−0.038 (3)	−0.005 (3)

### Geometric parameters (Å, °)

**Table:**

Cr—O15	1.912 (3)	N24—H24B	0.890
Cr—O11	1.963 (3)	N24—H24C	0.890
Cr—O13	1.966 (4)	C11—C15	1.522 (7)
Cr—O1	1.988 (4)	C11—C14	1.533 (7)
Cr—N21	2.044 (4)	C11—C12	1.549 (7)
Cr—N23	2.071 (4)	C12—C13	1.514 (7)
O1—H1A	0.846	C12—H12A	0.970
O1—H1B	0.858	C12—H12B	0.970
O11—C13	1.287 (6)	C15—C16	1.512 (7)
O12—C13	1.233 (6)	C15—H15A	0.970
O13—C14	1.292 (6)	C15—H15B	0.970
O14—C14	1.234 (6)	C21—C22	1.334 (7)
O15—C11	1.424 (6)	C21—C24	1.456 (7)
O16—C16	1.241 (7)	C22—S21	1.716 (6)
O17—C16	1.256 (7)	C22—H22	0.9300
N21—C23	1.328 (6)	C23—S21	1.733 (6)
N21—C21	1.405 (7)	C24—C25	1.334 (8)
N22—C23	1.314 (7)	C25—S22	1.721 (6)
N22—H22A	0.849	C25—H25	0.9300
N22—H22B	0.837	C26—S22	1.731 (6)
N23—C26	1.321 (6)	O1W—H1WB	0.839
N23—C24	1.399 (7)	O1W—H1WA	0.971
N24—C26	1.328 (7)	O2W—H2WA	0.966
N24—H24A	0.890	O2W—H2WB	0.870
			
O15—Cr—O11	90.51 (14)	C13—C12—H12A	108.6
O15—Cr—O13	83.15 (15)	C11—C12—H12A	108.6
O11—Cr—O13	88.35 (15)	C13—C12—H12B	108.6
O15—Cr—O1	94.33 (15)	C11—C12—H12B	108.6
O11—Cr—O1	88.97 (15)	H12A—C12—H12B	107.6
O13—Cr—O1	176.30 (16)	O12—C13—O11	121.9 (5)
O15—Cr—N21	96.93 (16)	O12—C13—C12	117.5 (5)
O11—Cr—N21	172.51 (16)	O11—C13—C12	120.6 (4)
O13—Cr—N21	91.64 (16)	O14—C14—O13	124.7 (5)
O1—Cr—N21	91.36 (16)	O14—C14—C11	120.7 (5)
O15—Cr—N23	172.24 (16)	O13—C14—C11	114.5 (5)
O11—Cr—N23	93.22 (15)	C16—C15—C11	112.2 (5)
O13—Cr—N23	90.15 (17)	C16—C15—H15A	109.2
O1—Cr—N23	92.53 (17)	C11—C15—H15A	109.2
N21—Cr—N23	79.28 (16)	C16—C15—H15B	109.2
Cr—O1—H1A	125.5	C11—C15—H15B	109.2
Cr—O1—H1B	113.5	H15A—C15—H15B	107.9
H1A—O1—H1B	119.2	O16—C16—O17	124.3 (6)
C13—O11—Cr	129.5 (3)	O16—C16—C15	118.1 (6)
C14—O13—Cr	111.3 (3)	O17—C16—C15	117.6 (5)
C11—O15—Cr	107.2 (3)	C22—C21—N21	114.6 (5)
C23—N21—C21	110.7 (4)	C22—C21—C24	130.5 (5)
C23—N21—Cr	133.6 (4)	N21—C21—C24	114.8 (4)
C21—N21—Cr	115.7 (3)	C21—C22—S21	111.5 (5)
C23—N22—H22A	97.9	C21—C22—H22	124.2
C23—N22—H22B	143.4	S21—C22—H22	124.2
H22A—N22—H22B	117.1	N22—C23—N21	123.3 (5)
C26—N23—C24	110.4 (5)	N22—C23—S21	123.2 (4)
C26—N23—Cr	134.0 (4)	N21—C23—S21	113.5 (4)
C24—N23—Cr	115.1 (3)	C25—C24—N23	115.5 (5)
C26—N24—H24A	109.5	C25—C24—C21	130.1 (5)
C26—N24—H24B	109.5	N23—C24—C21	114.4 (5)
H24A—N24—H24B	109.5	C24—C25—S22	110.7 (4)
C26—N24—H24C	109.5	C24—C25—H25	124.7
H24A—N24—H24C	109.5	S22—C25—H25	124.7
H24B—N24—H24C	109.5	N23—C26—N24	124.5 (5)
O15—C11—C15	110.0 (4)	N23—C26—S22	113.8 (4)
O15—C11—C14	109.6 (4)	N24—C26—S22	121.7 (4)
C15—C11—C14	110.7 (4)	C22—S21—C23	89.6 (3)
O15—C11—C12	108.6 (4)	C25—S22—C26	89.6 (3)
C15—C11—C12	110.0 (4)	H1WB—O1W—H1WA	108.0
C14—C11—C12	107.9 (4)	H2WA—O2W—H2WB	107.4
C13—C12—C11	114.5 (4)		

### Hydrogen-bond geometry (Å, °)

**Table:**

*D*—H···*A*	*D*—H	H···*A*	*D*···*A*	*D*—H···*A*
O1—H1A···O2W	0.85	1.85	2.689 (7)	171
O1—H1B···O12^i^	0.86	1.76	2.597 (5)	164
O1W—H1WB···O15^ii^	0.84	1.92	2.718 (7)	159
O1W—H1WA···O14	0.97	1.83	2.782 (7)	168
O2W—H2WA···O13^iii^	0.97	1.85	2.781 (6)	160
O2W—H2WB···O16^iv^	0.87	1.89	2.690 (8)	152
N22—H22A···O15	0.85	2.12	2.942 (6)	162
N22—H22B···O1W^v^	0.84	2.15	2.867 (7)	144
N24—H24A···O11	0.89	2.05	2.864 (6)	152
N24—H24B···O14^vi^	0.89	2.11	2.901 (6)	148

Symmetry codes: (i) −*x*+1, −*y*+1, −*z*+1; (ii) *x*−1, *y*, *z*; (iii) *x*+1, *y*, *z*; (iv) −*x*+1, −*y*, −*z*+2; (v) −*x*, −*y*, −*z*+2; (vi) −*x*, −*y*+1, −*z*+1.
